# An Integrated ddPCR Lab-on-a-Disc Device for Rapid Screening of Infectious Diseases

**DOI:** 10.3390/bios14010002

**Published:** 2023-12-21

**Authors:** Wanyi Zhang, Lili Cui, Yuye Wang, Zhenming Xie, Yuanyuan Wei, Shaodi Zhu, Mehmood Nawaz, Wing-Cheung Mak, Ho-Pui Ho, Dayong Gu, Shuwen Zeng

**Affiliations:** 1 Department of Biomedical Engineering, The Chinese University of Hong Kong, Shatin, Hong Kong SAR 999077, China; wyzhang@link.cuhk.edu.hk (W.Z.); 1155066378@link.cuhk.edu.hk (Z.X.); wei-yy18@link.cuhk.edu.hk (Y.W.); shaodizhu@link.cuhk.edu.hk (S.Z.); mehmoodnawaz@cuhk.edu.hk (M.N.); wing.cheung.mak@cuhk.edu.hk (W.-C.M.); 2 School of Public Health, Guangdong Medical University, Dongguan 523808, China; leee118@gdmu.edu.cn; 3 Laboratory Medicine, Shenzhen Key Laboratory of Medical Laboratory and Molecular Diagnostics, Shenzhen Institute of Translational Medicine, The First Affiliated Hospital of Shenzhen University, Shenzhen Second People’s Hospital, Shenzhen 518035, China; wanhood@email.szu.edu.cn; 4 Key Laboratory of Optoelectronic Devices and Systems of Ministry of Education and Guangdong Province, College of Physics and Optoelectronic Engineering, Shenzhen University, Shenzhen 518060, China; yuyewang@szu.edu.cn; 5 Light, Nanomaterials & Nanotechnologies (L2n), CNRS-EMR 7004, Université de Technologie de Troyes, 10000 Troyes, France

**Keywords:** lab-on-a-disc (LOAD), digital droplet PCR, inspection of infectious disease, clinical detection, point-of-care (POC)

## Abstract

Digital droplet PCR (ddPCR) is a powerful amplification technique for absolute quantification of viral nucleic acids. Although commercial ddPCR devices are effective in the lab bench tests, they cannot meet current urgent requirements for on-site and rapid screening for patients. Here, we have developed a portable and fully integrated lab-on-a-disc (LOAD) device for quantitively screening infectious disease agents. Our designed LOAD device has integrated (i) microfluidics chips, (ii) a transparent circulating oil-based heat exchanger, and (iii) an on-disc transmitted-light fluorescent imaging system into one compact and portable box. Thus, droplet generation, PCR thermocycling, and analysis can be achieved in a single LOAD device. This feature is a significant attribute for the current clinical application of disease screening. For this custom-built ddPCR setup, we have first demonstrated the loading and ddPCR amplification ability by using influenza A virus-specific DNA fragments with different concentrations (diluted from the original concentration to 10^7^ times), followed by analyzing the droplets with an external fluorescence microscope as a standard calibration test. The measured DNA concentration is linearly related to the gradient–dilution factor, which validated the precise quantification for the samples. In addition to the calibration tests using DNA fragments, we also employed this ddPCR-LOAD device for clinical samples with different viruses. Infectious samples containing five different viruses, including influenza A virus (IAV), respiratory syncytial virus (RSV), varicella zoster virus (VZV), Zika virus (ZIKV), and adenovirus (ADV), were injected into the device, followed by analyzing the droplets with an external fluorescence microscope with the lowest detected concentration of 20.24 copies/µL. Finally, we demonstrated the proof-of-concept detection of clinical samples of IAV using the on-disc fluorescence imaging system in our fully integrated device, which proves the capability of this device in clinical sample detection. We anticipate that this integrated ddPCR-LOAD device will become a flexible tool for on-site disease detection.

## 1. Introduction

In recent years, the increase in emerging infectious diseases has presented a significant challenge to global public health [1,2]. The polymerase chain reaction (PCR) method has been the gold standard for detecting genes since 1983 [3,4]. Quantitative real-time PCR (qPCR) is currently the mainstream technology for nucleic acid molecular detection of pathogens. However, qPCR relies on standard curves or reference genes to determine the nucleic acid quantity, and it is only capable of qualitative detection. Moreover, its sensitivity and precision are limited when detecting low-abundance targets or identifying minor differences in template concentrations.

Digital droplet PCR (ddPCR) has emerged as a third-generation nucleic acid detection technology, following end-point PCR and quantitative real-time PCR (qPCR). It is a quantitative analysis tool in precision medicine to conduct precise nucleic acid detection [5,6]. By diluting the concentration of target molecules to a sufficiently low level, ddPCR facilitates the enumeration of positive reaction chambers. This is accomplished by distributing the attenuated target molecules across independent reaction chambers to enable single-molecule amplification. ddPCR offers several advantages over qPCR, including high-throughput, sensitivity, specificity, and precision. It has a unique advantage in enabling the exact counting of nucleic acid copies without relying on standard curves [7]. This feature allows for direct and independent DNA quantification, providing more precise and reproducible data with a strong anti-interference ability [8]. Furthermore, ddPCR has proven to be an effective method for detecting low-abundance targets [9], which holds great potential in pathogen detection applications [5,10,11,12,13]. The emergence of ddPCR has greatly improved the accuracy and sensitivity of nucleic acid detection. 

Despite the advantages of droplet digital PCR (ddPCR) in quantitatively detecting nucleic acids, the adoption of this technology remains limited due to the high costs and complex operating procedures involved. Taking the commercial droplet digital PCR platform QX200 ddPCR system from Bio-Rad as an example, it has been introduced to the market for applications such as early cancer diagnosis, prenatal diagnosis, and single-cell analysis [14]. The platform utilizes microfluidics technology to isolate liquids into independent reaction chambers or produce uniform water-in-oil microemulsions for parallel reactions. However, the system requires separate containers and machines for droplet generation, PCR amplification, and signal collection [15]. This separate apparatus working scheme leads to sample loss and detection signal variations. Additionally, the inflexibility of the protocol and reaction system also limits researchers’ ability to customize the assays to their own needs.

Centrifugal microfluidics turns out to be a promising solution for highly integrated and efficient sample manipulation of a point-of-care (POC) device. Centrifugal microfluidics has the capability of integrating multiple unit operations, including valve control, liquid transfer, quantification, mixing, measurement, separation, etc., into one compact disc [16,17,18]. In centrifugal platforms, only a motor is needed to provide the centrifugal force for sample manipulation on a disc. No external pumps or valves are needed. Moreover, multiplexed detection can be easily achieved, since all points on the disc are in the centrifugal force field. This centrifugal-driven approach offers the advantages of compactness, highly parallel batch processing, and the inherent availability of density-based sample separation [16]. These merits of centrifugal microfluidics provide great opportunities for integrating a variety of complicated functions into one device for POC diagnosis. Additionally, the convenient integration with optoelectronic detection technology has given microfluidic platforms broad prospects in many complex applications, such as fluorescence detection, electrochemical detection, protein calibration measurement, polymerase chain reaction, etc. [19,20]. Overall, the undemanding droplet generation operations and compatibility with various functions integrated on the disc simplify the detection process and allow even nonprofessionals to use the system directly [21]. These advantages have made the LOAD platform an excellent choice for rapid on-site disease detection. 

Although some different POC nucleic acid testing devices have been reported [22,23,24], there are still some challenges to be addressed for the existing devices and platforms: (i) low integration and reliance on specialized equipment. (ii) bulky size, and (iii) high requirements for professionals. Scientists are thus continuing to explore the development of integrated, portable, and user-friendly ddPCR platforms [21,25,26]. For example, Schuler et al. have already made progress with their ddPCR on-disc system [20], which significantly reduces the need for manual operations. To further integrate the digital nucleic acid detection platform, various research groups have introduced portable POC devices based on the ddPCR method. Recently, Men’s group introduced a low-cost microfluidic adaptive printing (MAP-ddPCR) system [27], and Li’s group created a novel ddPCR microfluidic chip that integrates all main functions of ddPCR assays, including droplet creation, PCR amplification, and fluorescence detection [28]. However, these innovative systems still rely on a commercial thermal cycler or an external fluorescence imager. Moreover, the current devices in this field are based on isothermal nucleic acid amplification methods such as RPA and LAMP [29,30,31,32]. Despite their advantages in rapid detection, affordability, and ease of use, these isothermal methods are relatively new compared to PCR. They have not yet matured enough to replace PCR as the gold standard for disease detection. Therefore, it is crucial to develop a fully integrated, portable, and user-friendly ddPCR-LOAD device to achieve simultaneously built-in droplet generation, PCR thermocycling, and on-disc fluorescence detection, which can be used for effective nucleic acid screening.

In this paper, we proposed an integrated ddPCR device based on a centrifugal lab-on-a-disc system that enables “sample-to-answer” operations. The device is designed to enable automated on-disc droplet generation. These droplets align in the PCR thermocycling and readout zone by capillary action. The target gene in the microdroplet is amplified on the disc through thermocycling based on a circulating oil-based heat exchange system. On-disc image recording and analysis are available by integrating an on-disc fluorescent imaging system. This all-in-one ddPCR-LOAD device is capable of measuring the absolute amount of molecular genetic products. Thus, this portable device will provide an on-site screening test solution for disease diagnosis. We anticipate that it will become a valuable tool for various molecular biology applications, including detecting pathogens, analyzing gene expression, and measuring disease-causing mutations.

## 2. Materials and Methods

### 2.1. Samples Collection and Preparation

Four infectious viral plasmids and five human infectious samples were used as amplification templates in this work (SI, Table S1). The four types of viral plasmids were influenza A virus (IAV), respiratory syncytial virus (RSV), scarlet fever (SF), and dengue virus type I (DENV-I). The five clinical samples were from patients infected by different viruses. They were influenza A virus (IAV), respiratory syncytial virus (RSV), varicella zoster virus (VZV), Zika virus (ZIKV), and adenovirus (ADV). All clinical samples were collected according to the standard clinical sample collection protocols, with ethical declaration. For the viral plasmid DNA, it was prepared in a serial dilution of the stock concentration (diluted from the original concentration up to 10^7^ times). For the human clinical samples, nucleic acid extraction was carried out using the Viral RNA Kit (Catalog No. 11858882001, Roche, Boston, MA, USA) for the above five positive clinical samples, and then, the extracted RNA was reverse-transcribed into cDNA using PrimeScript™ RT Master Mix (Catalog No. RR036A, Takara, Japan).

The PCR reagent used in this study was ddPCR™ EvaGreen Supermix (Catalog No. 1864033, Bio-Rad Laboratories Inc., Hercules, CA 94547, USA), and the EvaGreen based-PCR experiment was conducted according to the standard protocol. The EvaGreen-based PCR reaction mixture was assembled from a 2 × ddPCR™ EvaGreen Supermix (Bio-Rad Laboratories Inc., Hercules, CA 94547, USA), primers, and template (variable volumes, DNA reserved-transcribed from clinical samples or series-diluted viral plasmid DNA) in a final volume of 20 μL. The detailed sequences of the primer pairs used in this study can be found in the Supporting Information (SI, Table S1).

### 2.2. Workflow of ddPCR

**Figure 1:**
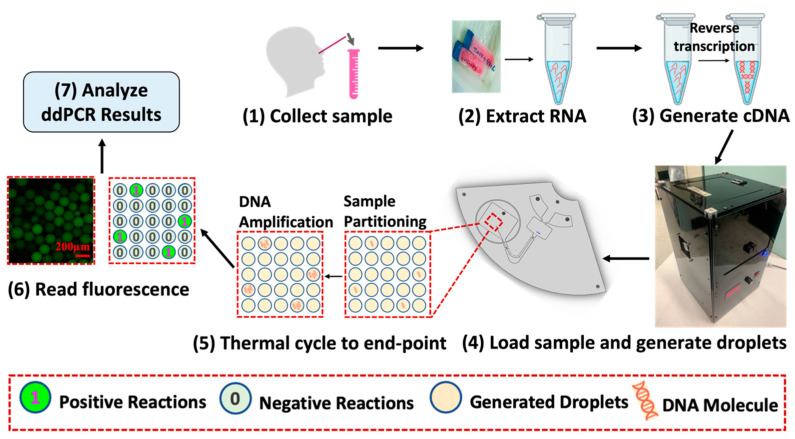
Sample processing and droplet digital PCR workflow. The ddPCR workflow includes sample collection, RNA extraction, and cDNA synthesis, followed by droplet generation and amplification on the ddPCR-LOAD device. Fluorescence images were captured for the results analysis.

The workflow of ddPCR is illustrated in Figure 1. First, samples were collected, followed by the extraction of RNA and the synthesis of cDNA. Next, as shown in step 4, the synthesized cDNA sample was loaded onto the ddPCR-LOAD device and divided into multiple independent partitions by droplet generation. These droplets contained either a limited number of target DNA molecules or none [33]. The droplets were then amplified by the on-disc thermocycling system (transparent circulating oil-based heat exchanger). After PCR thermocycling to the end point, fluorescent signals were detected in droplets that contained amplified target sequences, followed by some calibration tests using a Leica fluorescence microscope. Moreover, we demonstrated the capability of the on-disc fluorescence imaging system in our fully integrated device. Finally, fluorescence images were used for the subsequent analyses. The results of the PCR amplification were represented in binary form as either “1” or “0”, visualized by the presence or absence of fluorescent signals. By counting the number of fluorescent droplets, we could quantitatively measure the concentration of target DNA.

### 2.3. Design of the Integrated ddPCR-on-a-Disc Device

#### 2.3.1. On-Disc Fluorescence Imaging System

**Figure 2:**
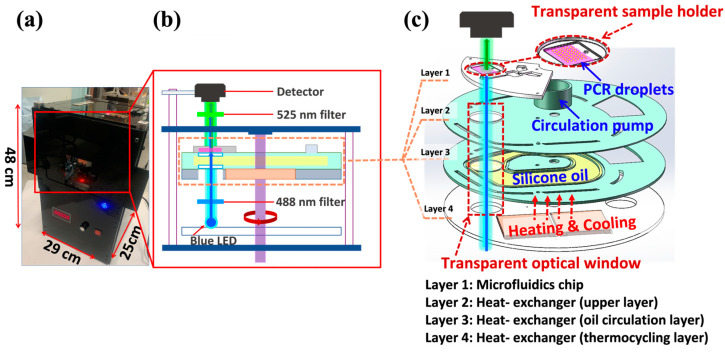
Physical and schematic diagram of the integrated ddPCR-LOAD device. (**a**) A photograph of the integrated ddPCR-on-disc device is shown with dimensions of 290 mm × 250 mm × 480 mm. (**b**) A schematic illustration of the device, which includes a centrifugal lab-on-a-disc (LOAD) subsystem and a fluorescent imaging subsystem. (**c**) Detailed diagram of the LOAD subsystem. The LOAD subsystem encompasses a microfluidics chip (layer 1) and a transparent circulating oil-based heat exchanger (layers 2–4).

Figure 2 illustrates the schematic layout of the ddPCR-LOAD device. The size of this portable device is 290 mm × 250 mm × 480 mm (see Figure 2a). The integrated device consists primarily of two components, as shown in Figure 2b: a fluorescence imaging subsystem (vertical part) and a spinning lab on-a-disc (LOAD) subsystem (horizontal part). The images of the device are shown in the Supplementary Information for demonstrating the operation of the system (Figure S1). The on-disc fluorescence imaging system contains four main parts: a light source (blue LED), a 488 nm excitation filter (FBH488-10, Thorlabs Inc., Newton, NJ 07860, USA), a 525 nm emission filter (MF525-39, Thorlabs Inc., Newton, NJ 07860, USA), and a camera (MU500B, AmScope, Irvine, CA 92606, USA). The built-in light source is positioned beneath the LOAD subsystem, and it illuminates the ddPCR sample through an excitation filter. The fluorescent sample was imaged by the camera placed at the opposite side of LOAD subsystem after an emission filter. This approach has the advantage of eliminating the need for sophisticated optical structures, thus simplifying the integration process, which results in cost reduction. Moreover, to enable the light source to reach the sample area of the microfluidic chip directly through the heating elements, a novel microfluidic chip with a PDMS–quartz composite structure is developed for the LOAD subsystem. Additionally, a transparent optical window is incorporated into the circulating oil-based heat exchange system under the microfluidic chip layer (purple area located in the horizontal part in Figure 2b), which allows for on-disc fluorescence imaging. This makes the system more portable and easier to be integrated into a compact device for on-site ddPCR analysis.

#### 2.3.2. Transparent Circulating Oil-Based Heat Exchanger

The detailed design of the microfluidics chip (layer 1) and the transparent circulating oil-based heat-exchanging disc (layers 2–4) is shown in Figure 2c and Figure S1B. The circulating oil-based heat-exchanging disc is composed of three layers: (i) the upper layer (layer 2), which is in direct contact with the microfluidics chip (layer 1) and also serving as the disc holder; (ii) the middle layer, which provides a channel for silicone oil circulation (layer 3); and (iii) the bottom layer, which holds two thermoelectric coolers (TECs) for providing PCR thermocycling (layer 4). The circulation pump provides the power source for the oil circulation, which is placed in layer 2. It consists of a three-phase DC brushless motor and a customized fan blade fabricated by 3D printing techniques with high-temperature resistant nylon material. Layer 3 is filled with heat-conducting oil (low-viscosity dimethyl silicone) for improved heat conductivity and liquid fluidity. The combination of the three-phase DC brushless motor and fan blade with the heat-conducting oil results in an efficient and reliable heat transfer system that ensures rapid heating and cooling.

During the PCR thermocycling process, the oil-based heat exchanger serves to circulate the heat-conductive oil in a clockwise direction inside the flow channel. The two TECs mounted in layer 4 can heat up and cool down the silicone oil, which is then transferred to the transparent sample holder by circulating, allowing for precise temperature control in the sample zone (layers 2–4, Figure 2c). The closed-loop workflow of the on-disc thermal controlling is illustrated in Figure S2A. The temperature sensor collects real-time data, and based on these data, the temperature control system cooperates with the PID algorithm to accurately control the TECs for bidirectional temperature control. The three layers of the heat exchanger work together to create a programmable thermocycling system that ensures accurate temperature control and prevents temperature instability. The temperature curve for PCR (4 cycles out of 42 cycles) is illustrated in Supporting Information Figures S2B and S3. To measure the actual temperature of the reagent, a thermocouple is inserted from the left inlet of the microfluidic chip. This thermocouple serves as an external sensor for temperature calibration. The temperature curve demonstrates that the device achieves the following performance: an average heating rate of 0.25 °C/s and an average cooling rate of 0.28 °C/s, resulting in a PCR temperature cycling duration of 6 min and 40 s for each cycle. These performance characteristics meet the requirements for conventional or ddPCR experiments. As a consequence, the design of this circulating oil-based heat exchanger provides an effective way to regulate the temperature for ddPCR experiments and enables the following transmission-based fluorescence detection. 

#### 2.3.3. Microfluidic Chip for Droplet Generation

**Figure 3:**
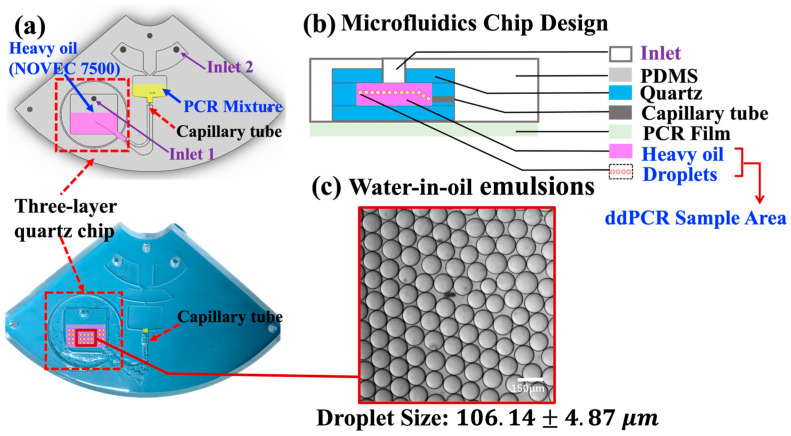
(**a**)The schematic diagram and the image of a PDMS–glass microfluidic chip. (**b**) Illustration of the design of the microfluidics chip for the ddPCR bioassay. (**c**) Bright field microscopy image of droplets generated with EvaGreen ddPCR mix in the sample region in Figure 3a (marked red-dotted rectangle). Data represent the mean ± SD, with n = 100.

The microfluidic chip consists of two main parts: a PDMS-based polymer holder and a three-layer quartz chip (highlighted with red, dotted box in Figure 3a). The PDMS-based polymer holder was designed using SolidWorks and fabricated with computer numerical control milling. The mold was designed using SolidWorks and fabricated with a digital controlled engraver. Then, a 10:1 (*w*/*w*) mixture of the polydimethylsiloxane (PDMS) prepolymer and curing agent (Dow Corning SYLGARD184, USA) was poured into the milled mold, incubated at 65 °C for 2 h until solid, and formed a structured microfluidic holder. Within the disc layout, a round shape well was left open for embedding the three-layer quartz chip. Moreover, the centrifugal microfluidic chip was designed based on “step emulsification” geometry, which can generate droplets with defined sizes without any external pumps or valves, as reported in the literature [16]. The V-shaped channel remains open for PDMS refilling in order to mount the capillary elements tightly within the chip. The final chip is sealed with a layer of optical transparent adhesive film (Life Technologies, Waltham, MA, USA).

### 2.4. ddPCR Screening Test for Clinical Samples

After sample preparation (i.e., (i) sample collection, (ii) RNA extraction, and (iii) cDNA generation, illustrated in Section 2.1), the PCR mixture proceeded with droplet generation in the microfluidic chip, 20 µL of PCR reaction mixture (illustrated in Section 2.1) was loaded into inlet 2 of the microfluidic chip (highlighted in the yellow area in Figure 3a), and 70 µL droplet generation oil for EvaGreen (Catalog No. 1864005, Bio-Rad Laboratories Inc., Hercules, CA 94547, USA) was loaded into inlet 1 of the chip (highlighted in the purple area, Figure 3a). The designed microfluidic chip could generate droplets of defined diameters by controlling the rotational speed. In this work, all droplets were generated under a rotating speed of 2000 rpm. The droplets then went through a customized capillary to the sample region. Next, the target gene was amplified to the end point and detected directly on the integrated ddPCR system. The thermal cycling conditions for the PCR consisted of an initial incubation at 95 °C for 5 min, followed by 42 cycles of denaturation at 95 °C for 15 s, annealing at 55 °C for 15 s, and extension at 72 °C for 30 s. The reaction was then subjected to a final step at 72 °C for 5 min. Finally, a Leica fluorescence microscope or on-disc fluorescent imaging subsystem was utilized to capture the fluorescence images, revealing the amplified gene contained in the droplet. The fluorescence images were then analyzed with MATLAB R2019b software. The absolute quantification of the sample was calculated based on the image analysis by Poisson distribution [34] (shown in the Supporting Information).

## 3. Results and Discussion

### 3.1. Droplet Generation

The compact disposable microfluidic chip consisted of two main layers: a PDMS-based holder and a three-layer glass chip embedded in the round sample area, as shown in Figure 3a. The centrifugal microfluidics chip is designed based on “step emulsification” geometry, which can generate droplets with defined sizes by pump-free fluidic actuation. The size of the droplets depends on the diameter of the capillaries and the spinning speed. This design integrates a commercially available capillary into microfluidic chips without using lithography techniques [24,35], which greatly simplifies the fabrication process and reduces the cost [16]. Droplet generation oil from EvaGreen (Bio-Rad Laboratories Inc., Hercules, CA 94547, USA) was used as the oil phase in the water-in-oil emulsion. As the LOAD system spins, the aqueous phase will be “cut off” by centrifugal forces, which generates droplets in the sample region. 

As shown in Figure 3b, a three-layer glass chip was embedded into a PDMS-based polymer holder to serve as a container for droplet generation and the subsequent PCR thermocycling process, which prevented evaporation issues caused by the porous structure of PDMS during thermocycling [20]. Figure 3c shows a uniform droplet size distribution with a diameter of 106.14 ± 4.87 μm under the spinning speed of 2000 rpm.

### 3.2. System Calibration

**Figure 4:**
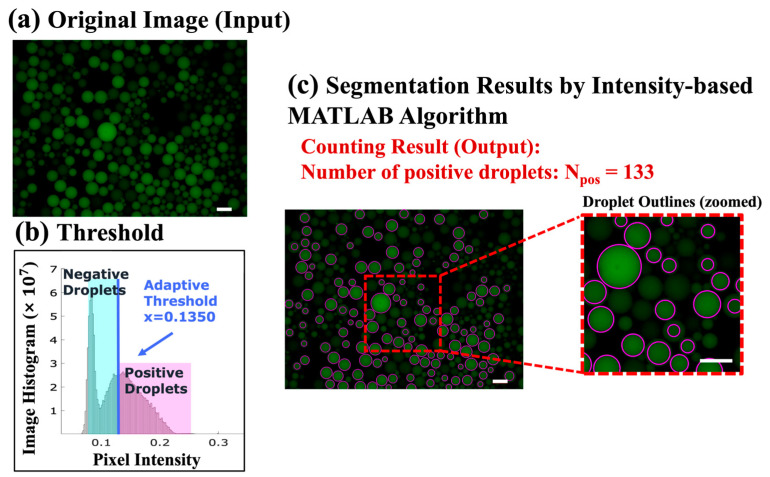
Image analysis using the MATLAB algorithm. (**a**) An original image extracted from the experimental results of the IAV human infectious sample detection (1 out of 35 images), serving as the input to the algorithm. The scale bar represents 200 µm. (**b**) An image histogram displaying the adaptive threshold utilized for droplet classification and segmentation. (**c**) The counted number of positive (bright) droplets in Figure 4a is 133, indicating the result of droplet counting. The scale bar represents 200 µm.

In order to evaluate the performance of the proposed ddPCR-LOAD device, we conducted on-disc ddPCR experiments and used a fluorescence microscope (Leica CTR6500, Leica Microsystems, Wetzlar, Germany) for the system calibration. The resulting images were analyzed using MATLAB software, as illustrated in Figure 4. As an example, Figure 4a depicts one of the original images extracted from the experiment results of the IAV clinical specimens. The original images were used as input for the algorithm for droplet classification and segmentation. To this end, an image histogram, shown in Figure 4b, was generated to illustrate the threshold determination. The threshold was established based on the native function “gray thresh” in MATLAB software, which employs Otsu’s algorithm [36] to evaluate all potential values for the threshold between background and foreground. When the histogram exhibits no clear valleys or when the contrast between objects and the background is low, this method can successfully achieve the desired segmentation. The optimal threshold value was selected by minimizing the weighted sum of the variances within two clusters (“level = graythresh (image)”). In our study, the threshold for positive droplet segmentation was determined using the equation “Threshold = (1 + 1/6) * level”. This formula scales the calculated threshold level (obtained from the “graythresh” function) by a factor of (1 + 1/6) to set a slightly higher threshold value. This adjustment helps to ensure the segmentation of positive droplets.

For each image, the average intensity in each individual droplet was calculated. For example, in the sample image shown in Figure 4a, the calculated threshold value is x = (1 + 1/6) * level = 0.1350, as illustrated in Figure 4b. Finally, the resulting positive droplet segmentation and the corresponding number counting outcomes can be observed in Figure 4c. These results demonstrate the successful application of the thresholding technique for segmenting and counting positive droplets in the analyzed image.

**Figure 5:**
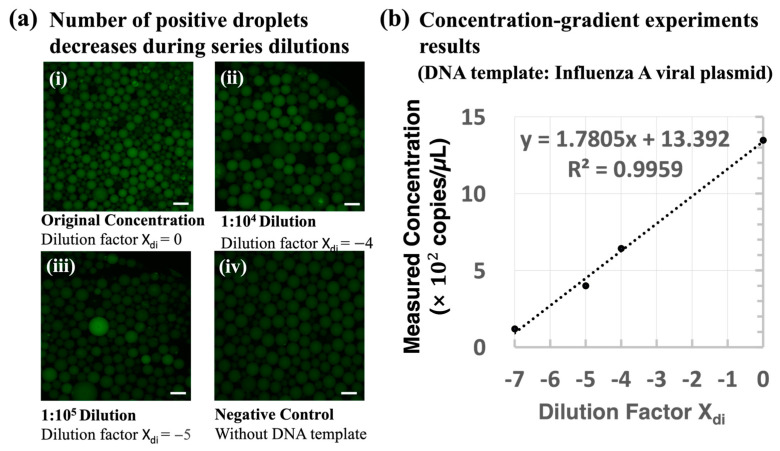
System calibration results from a series of dilution concentrations of IAV viral plasmid DNA. (**a**) Fluorescence distribution of droplets after ddPCR reactions at different cDNA concentrations. The target DNA used in this experiment is IAV viral plasmid DNA: (i) initial concentration (1 out of 30 images), (ii) diluted 10^4^ times (1 out of 19 images), (iii) diluted 10^5^ times (1 out of 14 images), and (iv) without a DNA template (1 out of 17 images). The scale bar represents 200 µm. (**b**) The measured concentration results were calculated based on the fluorescent droplet counting results, and the number of fluorescence droplets was detected using MATLAB software (see Figure 4). The linear fitting result R^2^ = 0.9959 shows the detected concentration results from the reported device were consistent with the gradient–dilution factor. The images were captured by a fluorescence microscope (Leica CTR6500) for system calibration.

The integrated system was calibrated using a serial dilution of the stock IAV viral plasmid DNA, spanning from 1:10^0^ to 1:10^7^. Next, fluorescence images of the droplets were taken with a Leica CTR6500 fluorescence microscope (Leica Microsystems, Germany) to capture the amplified gene contained in the droplet. Figure 5a shows the concentration–gradient results from the following experiments: (a) the initial concentration, (b) 10^4^ times diluted, (c) 10^5^ times diluted, and (d) without the DNA template. As the concentration of the template decreased, fewer positive droplets were observed in the images, which confirms the reliability of the LOAD disc for droplet generation and conducting thermocycling ddPCR experiments of varied concentrations. Figure 5b indicates the correlation between the measured concentration and the dilution factor X_di_ (= −log (dilution times), Figure 5a,b). In this figure, the measured concentrations were calculated based on the image analysis results obtained by MATLAB (2019b) (see Figure 4). Given the measured fraction of positive reactors, we calculated the initial concentration (measured concentration result) of the tested samples. The equations can be found in the Supporting Information. The results show that the measured concentration in the experiment has a good correlation with the gradient–dilution ratio (R^2^ = 0.9959), which verifies the capability of the droplet generation and PCR thermocycling functions of the proposed ddPCR LOAD device in the quantitation of gene targets. The concentration–gradient results of viral plasmid DNA have also proven the feasibility of quantitative measurements of this device for disease detection.

### 3.3. Detection of Human Infectious Samples

**Figure 6:**
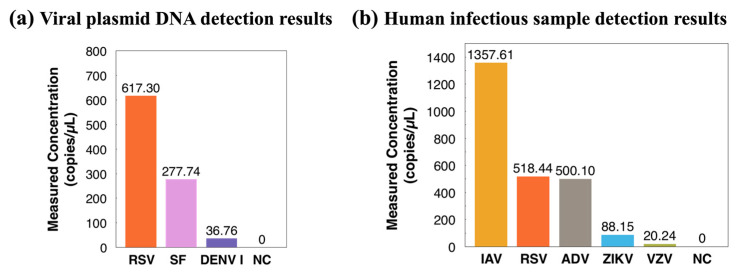
(**a**) Concentration measurements of three viral plasmid DNA on the ddPCR LOAD device (template: infectious virus plasmid). (**b**) Detection results of five human infectious samples on the ddPCR LOAD device. Abbreviations: RSV, respiratory syncytial virus; SF, scarlet fever; DENV-I, dengue virus type I; IAV, influenza A virus; ADV, adenovirus; ZIKV, Zika virus; VZV, varicella zoster virus, and NC, negative control.

Moving forward, three viral plasmid DNA samples were tested in Figure 6. These samples were injected into the reported ddPCR-LOAD device for droplet generation and PCR thermocycling and subsequently imaged using a Leica fluorescence microscope. Viral plasmids DNA are purified plasmids containing different specific viral gene sequences, including RSV, SF, and DENV-I. All three positive samples were successfully detected, and the concentration of each sample was measured to be 617.30, 277.74, and 36.76 copies/µL, respectively. Furthermore, to push our work towards clinical applications, five real human infectious samples were detected, including IAV, RSV, ADV, ZIKV, and VZV. We extracted RNA directly from clinical samples with unknown virulence gene abundance and reverse-transcribed them into cDNA for the following ddPCR experiments on the on-disc ddPCR LOAD system for droplet generation and PCR thermocycling. The detected concentrations analyzed with an external fluorescence microscope from the five samples were 1357.61, 518.44, 500.10, 88.15, and 20.24 copies/µL, respectively (shown in Figure 6b). The minimum detected concentration in this work was 20.24 copies/µL. 

### 3.4. On-Site Infectious Disease Detection

**Figure 7:**
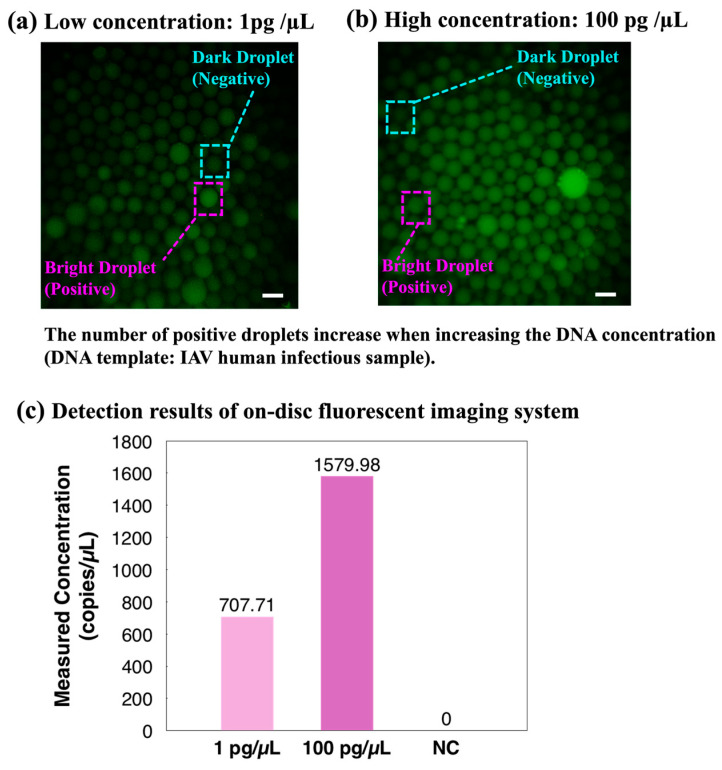
Proof-of-concept on-site validation of the integrated ddPCR-LOAD device. (**a**,**b**) Clinical droplet digital PCR based on different concentrations of the influenza A virus (IAV). The scale bar represents 200 µm. The number of positive droplets increases when increasing the DNA concentration. (**c**) Detection results of the on-disc fluorescent imaging system. Abbreviation: NC, negative control.

To further validate the integrated ddPCR-LOAD device, a proof-of-concept clinical sample detection experiment was demonstrated. After the on-disc ddPCR assays, fluorescent images were captured directly using the integrated fluorescent imaging system of the device. Two different concentration levels of the IAV clinical sample were injected into the device: 1 pg/µL and 100 pg/µL. The captured fluorescence images in Figure 7a,b clearly demonstrated an increase in the number of positive droplets with the increasing target DNA concentration. Subsequently, the quantitative concentrations of the two samples were calculated based on the image analysis results, as shown in Figure 7c. The results revealed that the two IAV clinical samples were detected as positive, and the measured concentration indicated the proposed device is capable of distinguishing clinical specimens with different concentration levels and quantifying target genes in on-site clinical samples.

## 4. Conclusions

In conclusion, we have developed an integrated ddPCR-LOAD device. This centrifugal force-driven device provides a portable, highly integrated, and user-friendly scheme for on-site pathogen detection. The centrifugal microfluidic chip is designed based on “step emulsification” geometry, which can generate droplets with defined sizes without any external pumps or valves. Moreover, a transmitted-light fluorescence system and transparent circulating oil-based heat exchanger were developed for better system integration. Based on this integrated device, we have successfully demonstrated ddPCR experiments for on-site infectious disease detection. The feasibility of this device was first validated by detecting IAV viral plasmid DNA templates at various concentration levels. The ddPCR experiments were conducted on the device and followed by analyzing the droplets using an external fluorescence microscope. Moving forward, five clinical infectious samples were quantitatively detected in this study. The minimum detected concentration in this work was found to be 20.24 copies/µL in the respiratory adenovirus samples (measured with an external fluorescence microscope). Finally, the proof-of-concept detection of clinical samples of IAV was demonstrated on the fully integrated device. Overall, this integrated ddPCR-LOAD device is capable of quantitative clinical sample detection. It has great potential in the early diagnosis of cancer, noninvasive prenatal diagnosis, single-cell analysis, high-throughput drug screening, and other biomedical applications.

## Data Availability

All data needed to evaluate the conclusions in the paper are present in the paper and/or the Supplementary Materials.

## Supplementary Materials

The following supporting information can be downloaded at https://www.mdpi.com/article/10.3390/bios14010002/s1: Table S1: Information of the 4 types of viral plasmid DNA and 5 human infectious samples used in this work. Figure S1: Equipment images. Figure S2: (A) The closed-loop workflow of the on-disc thermal controlling; (B) Illustration of the temperature curve for PCR inside the chamber, and the equations of the measured concentrations [34,37,38]. Figure S3: PCR thermocycling curve: (A) initial 4 cycles: cycle 1–cycle 4, (B) middle 4 cycles: cycle 21–cycle 24, and (C) final 4 cycles: cycle 39–cycle 42.
