# Supplementary material for: An Integrated ddPCR Lab-on-a-Disc Device for Rapid Screening of Infectious Diseases
Source: Biosensors (Basel). 2023 Dec 21;14(1):2. doi: 10.3390/bios14010002 (PMC10813669; doi:10.3390/bios14010002)
Supplement: Supplementary file 1 [file biosensors-14-00002-s001.zip › biosensors-2735838-supplementary.pdf]

Table S1: Information of the 4 types of viral plasmid DNA and 5 human infectious samples used in this work.

NO.	Virus Name	**Related Results**	Type	Forward Primer (5'-3')	Reverse Primer (5'-3')	Length (bp)
1	IAV	**Figure 5**	Plasmid	**ACAAGACCRATCYTGTCACCTCT**	**TTTAGGGCATTYTGGACAAAKCGTC**	150
2	RSV	**Figure 6A**	Plasmid	**TACTAATTTAGCTGGACATTGGAT**	**TTTCAAATTAATGAACATATGATCAG**	336
3	SF	**Figure 6A**	Plasmid	**ATACAACTGGAACAACATCCTACCTA**	**TACTGAAATACCAACATCAGCCA**	100
4	DENV- I	**Figure 6A**	Plasmid	**AGCCATACCCCCAACAGC**	**TGAGAAGCATGGTCACGGAT**	149
5	IAV	**Figure 6B, Figure 7**	Clinical Sample	**ACAAGACCRATCYTGTCACCTCT**	**TTTAGGGCATTYTGGACAAAKCGTC**	150
6	RSV	**Figure 6B**	Clinical Sample	**TACTAATTTAGCTGGACATTGGAT**	**TTTCAAATTAATGAACATATGATCAG**	336
7	ADV	**Figure 6B**	Clinical Sample	**CTCGGAGTACCTGAGTCCGG**	**CGTGGGATTTCTAAACTCATTTC**	100
8	ZIKV	**Figure 6B**	Clinical Sample	**AGGAGAAGCTGGGAAACCA**	**TTTTTTGACTCAGTGTCCTCTGA**	204
9	VZV	**Figure 6B**	Clinical Sample	**TCTCGTCCAATCACTACATCGT**	**AGGGAGTTCCAACAGTACTTAAAA**	301

Abbreviation: IAV, influenza A virus; RSV, respiratory syncytial virus; SF, scarlet fever; DENV-I, dengue virus type I; ADV,adenovirus; ZIKV, Zika virus, and VZV, varicella zoster virus.

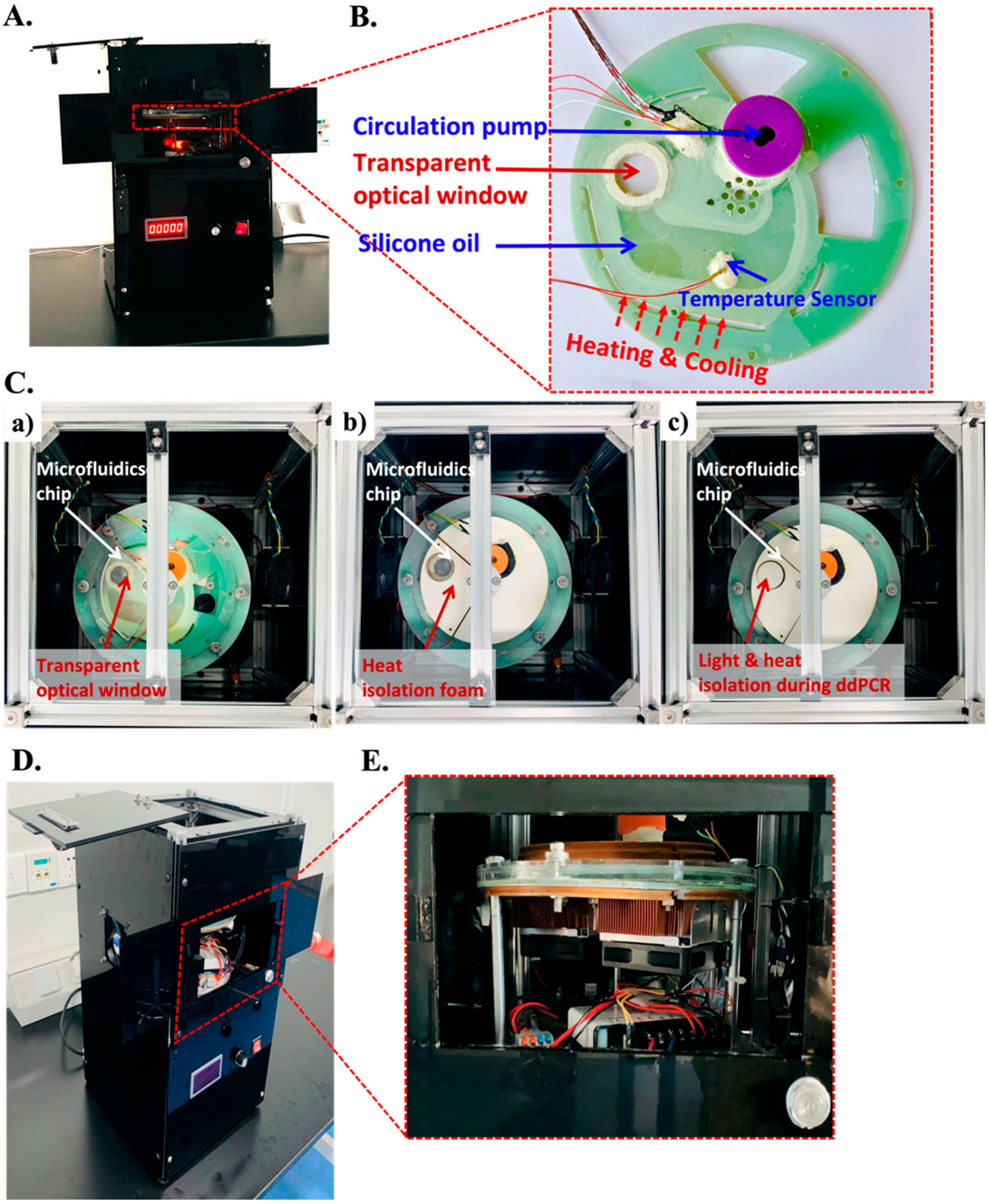

Figure S1. Equipment images: (A) A photograph of the integrated ddPCR-on-disc device and transparent circulating oil-based heat exchanger was highlighted with the red dotted box. (B) A photograph of the transparent circulating oil-based heat exchanger. (C) Top view of the device for showing the LOAD subsystem. (D-E) A photograph of the integrated ddPCR-on-disc device and the lab-on-a-disc (LOAD) subsystem was highlighted.

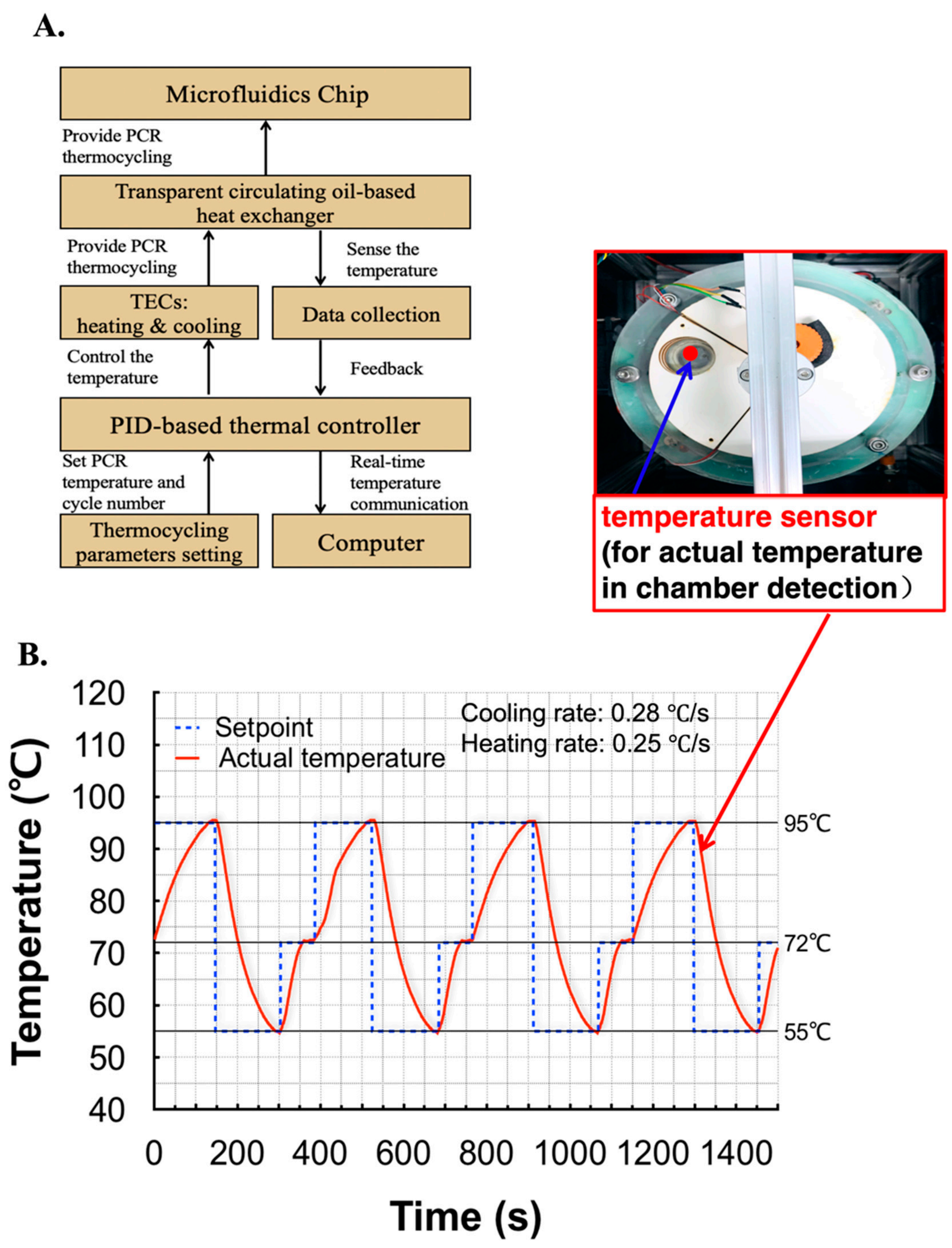

Figure S2. (A) The closed-loop workflow of the on-disc thermal controlling. (B) Illustration of the temperature curve for PCR inside chamber. The thermocouple was inserted from the left inlet of the microfluidic chip for measuring the real temperature of the reagent.

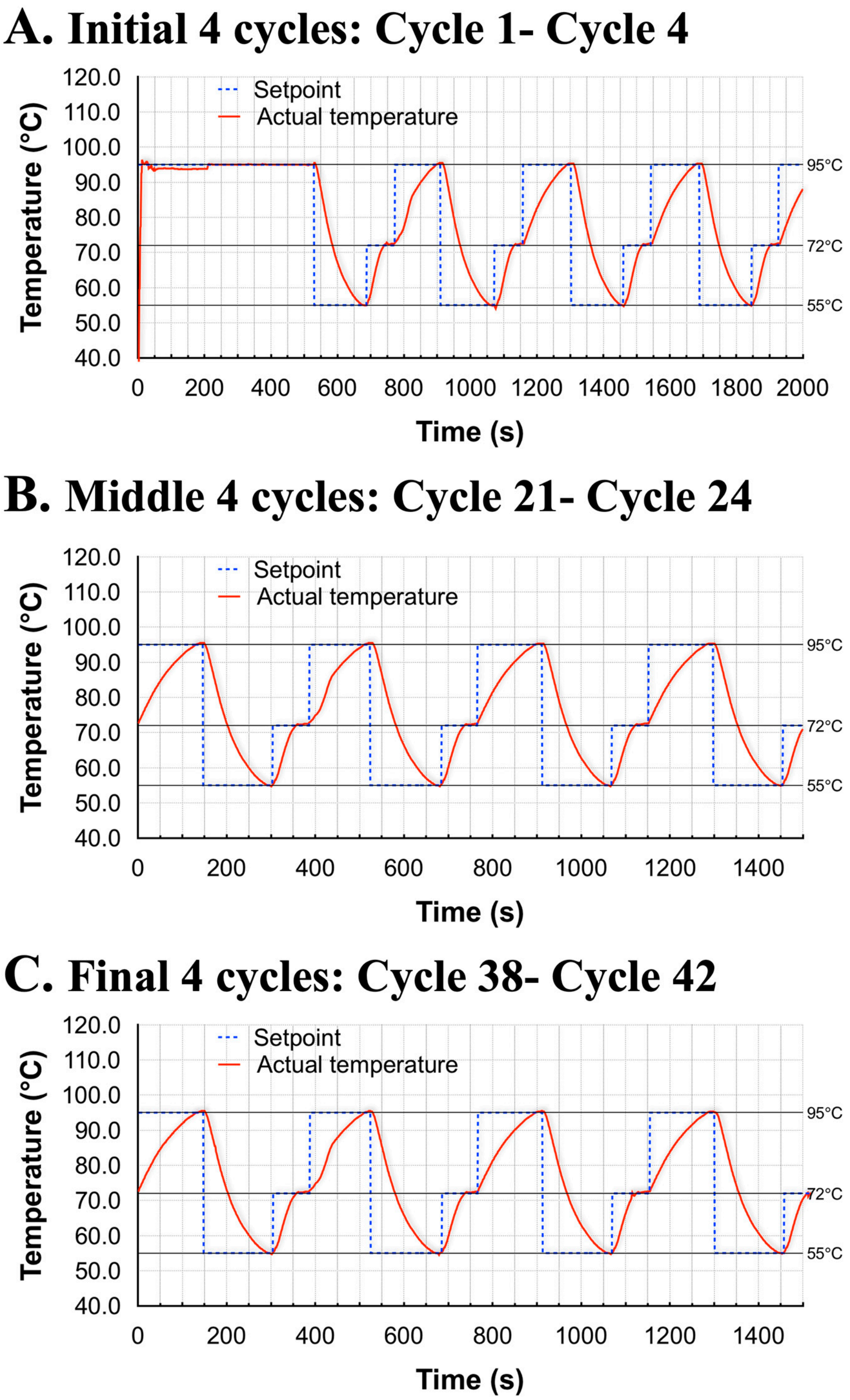

Figure S3: PCR Thermocycling Curve: (A) Initial 4 cycles: Cycle 1 - Cycle 4; (B) Middle 4 cycles: Cycle 21 - Cycle 24, and (C) Final 4 cycles: Cycle 38 - Cycle 42.

Equations of measured concentration:

In ddPCR experiments, each droplet is considered as an independent trial, and contains contains a discrete number (0, 1, 2, 3, etc.) of target biological entities (DNA molecules were used as templates in our work). The maximum number of DNA in each partition depends on the initial concentration of DNA templates. In order to quantify the concentration of samples (shown on the y-axis of Figure5B, Figure6A, Figure6B, and Figure7C) accurately, the results should be corrected mathematically by the Poisson distribution [1]. The Poisson distribution is a statistical distribution that describes the number of events that occur in a fixed interval of time or space. These events occur independently and at a constant rate [2]. In the context of ddPCR assays, the Poisson distribution is used to predict the number of droplets that contain DNA molecule by (s). The measured concentration, *C*, is estimated according to the following equations [3]:

$$C = -\frac{ln(1 - F_{pos})}{V} \quad (1)$$

$$F_{pos} = \frac{N_{pos}}{N_{total}} \quad (2)$$

In equation (1), the concentration *C* is defined as the number of target DNA molecules per droplet divided by the volume of each droplet *V*. The number of targets per droplet is predicted by $(-ln(1 - F_{pos}))$ using the Poisson distribution, where F_{pos} represents the fraction of positive droplets where target gene is presented. As shown in in equation (2), N_{pos} expresses the number of positive droplets and N_{total} is the number of total droplets.

References

1. A. S. Basu, *SLAS Technol.*, 2017, **22**, 369-386.
2. S. D. Poisson and S.-D. Poisson, *Recherches sur la probabilité des jugements en matière criminelle et en matière civile: précédées des règles générales du calcul des probabilités*, Bachelier, 1837.
3. K. R. Sreejith, C. H. Ooi, J. Jin, D. V. Dao and N. T. Nguyen, *Lab Chip*, 2018, **18**, 3717-3732.
